# Methylprednisolone Treatment in Brain Death-Induced Lung Inflammation–A Dose Comparative Study in Rats

**DOI:** 10.3389/fphar.2021.587003

**Published:** 2021-02-22

**Authors:** Judith E. Van Zanden, Nils A. ’T Hart, Petra J. Ottens, Bo Liu, Rolando A. Rebolledo, Michiel E. Erasmus, Henri G. D. Leuvenink

**Affiliations:** ^1^ Department of Surgery, University of Groningen, University Medical Center Groningen, Groningen, Netherlands; ^2^ Department of Pathology, University of Groningen, University Medical Center Groningen, Groningen, Netherlands; ^3^ Department of Neurosurgery, Tianjin Medical University General Hospital, Tianjin, China; ^4^ Institute for Medical and Biological Engineering, Schools of Engineering, Biological Sciences and Medicine, Pontificia Universidad Católica de Chile, Santiago, Chile; ^5^ Department of cardiothoracic surgery, University of Groningen, University Medical Center Groningen, Groningen, Netherlands

**Keywords:** brain death, lung donation, lung inflammation, methylprednisolone, donor management, dose comparison study, lung transplantation

## Abstract

**Background:** The process of brain death (BD) leads to a pro-inflammatory state of the donor lung, which deteriorates its quality. In an attempt to preserve lung quality, methylprednisolone is widely recommended in donor lung management. However, clinical treatment doses vary and the dose-effect relation of methylprednisolone on BD-induced lung inflammation remains unknown. The aim of this study was to investigate the effect of three different doses methylprednisolone on the BD-induced inflammatory response.

**Methods:** BD was induced in rats by inflation of a Fogarty balloon catheter in the epidural space. After 60 min of BD, saline or methylprednisolone (low dose (5 mg/kg), intermediate dose (12.5 mg/kg) or high dose (22.5 mg/kg)) was administered intravenously. The lungs were procured and processed after 4 h of BD. Inflammatory gene expressions were analyzed by RT-qPCR and influx of neutrophils and macrophages were quantified with immunohistochemical staining.

**Results:** Methylprednisolone treatment reduced neutrophil chemotaxis as demonstrated by lower IL-8-like CINC-1 and E-selectin levels, which was most evident in rats treated with intermediate and high doses methylprednisolone. Macrophage chemotaxis was attenuated in all methylprednisolone treated rats, as corroborated by lower MCP-1 levels compared to saline treated rats. Thereby, all doses methylprednisolone reduced TNF-α, IL-6 and IL-1β tissue levels. In addition, intermediate and high doses methylprednisolone induced a protective anti-inflammatory response, as reflected by upregulated IL-10 expression when compared to saline treated brain-dead rats.

**Conclusion:** We showed that intermediate and high doses methylprednisolone share most potential to target BD-induced lung inflammation in rats. Considering possible side effects of high doses methylprednisolone, we conclude from this study that an intermediate dose of 12.5 mg/kg methylprednisolone is the optimal treatment dose for BD-induced lung inflammation in rats, which reduces the pro-inflammatory state and additionally promotes a protective, anti-inflammatory response.

## Introduction

Despite developments in deceased after circulatory death (DCD) donation, donation after brain death (DBD) still serves as the major source for donor lungs (Cypel et al., 2015). In both DCD and DBD donors pathophysiological changes occur, which affect both quantity and quality of organs available for transplantation (Cooper et al., 1989; Neyrinck et al., 2013). Since only 30% of donor lungs are considered suitable for transplantation, lungs seem highly susceptible to damage. In contrast, over 70% of the abdominal organs are procured and utilized for transplantation (Rahmel, 2013). One of the mechanisms that detrimentally affects lung quality in DBD donors is activation of the immune system, which aggravates rejection of organs after transplantation (Avlonitis et al., 2003).

From the onset of brain death (BD), inflammatory mediators such as TNF-α, IL-1β, IL-6 and IL-8 are released by the ischemic brain (Wang et al., 1994; Wang et al., 1995; Liu et al., 1993; Liu et al., 1994). Local inflammation and the direct effect of the insult itself cause disruption of the blood-brain-barrier and brain stem death. Along with local activation of the immune system in the brain, a systemic inflammatory response syndrome (SIRS) occurs (McKeating et al., 1997; Lu et al., 2009). The peripheral immune system is activated and pro-inflammatory cytokines are released from the spleen, which augments the inflammatory response (Offner et al., 2006). Eventually, the BD-induced immunological mechanisms lead to a pro-inflammatory state of the potential donor lung, which is associated with increased rejection rates of transplanted donor lungs (Zweers et al., 2004).

Most clinical guidelines recommend administration of methylprednisolone in brain-dead lung donors, to promote hemodynamic stability of the donor and improve lung function after transplantation (Cooper et al., 1988; Follette et al., 1998). These treatment regimens vary from fixed single doses of 1–5 g methylprednisolone, to weight-base doses ranging from 15–60 mg/kg methylprednisolone (Dupuis et al., 2014). The effect of varying doses methylprednisolone on the BD-induced inflammatory status, has not been elucidated. The aim of this study was to investigate the effect of different doses methylprednisolone on the BD-induced inflammatory status of donor lungs. To this end, we subjected rats to BD and treated them with a low, intermediate or high dose methylprednisolone during donor management. We showed that an intermediate dose of 12.5 mg/kg methylprednisolone is the optimal treatment dose for BD-induced lung inflammation in rats.

## Materials and Methods

### Experimental Outline

**FIGURE 1:**
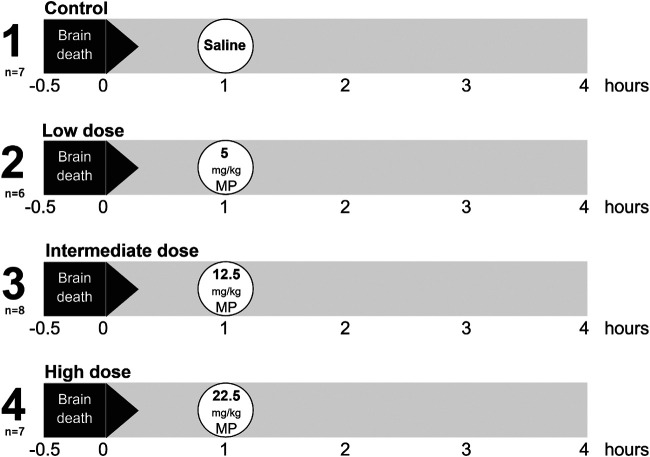
Experimental outline of the study. Rats were randomly assigned to one of four experimental groups, each consisting of 6-8 rats. Brain death (BD) was induced in all experimental groups, and 1 h after BD induction rats were treated intravenously with 1) 0.9% saline (control, n = 7), 2) low dose methylprednisolone (MP, 5 mg/kg of bodyweight (BW), n = 6), 3) intermediate dose methylprednisolone (12.5 mg/kg of BW, n = 8) and 4) high dose methylprednisolone (22.5 mg/kg of BW, n = 7).

Rats were randomly assigned to one of four experimental groups (6–8 rats/group, Figure 1). BD was induced in all experimental groups, and 1 h after BD induction rats were treated intravenously with 1) 0.9% saline (control, n = 7), 2) low dose methylprednisolone (5 mg/kg of bodyweight (BW), n = 6), 3) intermediate dose methylprednisolone (12.5 mg/kg of BW, n = 8) or 4) high dose methylprednisolone (22.5 mg/kg of BW, n = 7). The various doses of methylprednisolone administration were defined based on previous pilot experiments, in which 22.5 mg/kg methylprednisolone was the maximum dose in which hemodynamic stability was maintained.

### Rats

Male adult Fischer F344 rats (Harlan Netherlands B.V., Melderslo, The Netherlands) with a weight of 250–300 g were used. Rats received standard humane care in compliance with the Principles of Laboratory Animal Care (NIH Publication NO. 86-23, revised 1985) and the Dutch Law on Experimental Animal Care. Permission to conduct this study was approved by the local animal committee, according to the Experiments on Animals Act (Animal Welfare Body Utrecht, 2017).

### Rat Brain Death Model

BD was induced as previously described (Rebolledo et al., 2015). Rats were anesthetized by a mixture of 100% O_2_ and 5% isoflurane. The femoral vessels were cannulated to enable mean arterial pressure (MAP) measurements and fluid administration. Rats were tracheotomized, intubated with a 14G polyethylene tube and ventilated with tidal volumes of 2–3 ml/stroke. Respiratory rate was titrated throughout the experiment to maintain end-tidal CO_2_ between 20–22 mmHg, with a minimum frequency of 50/min and a maximum frequency of 120/min. Positive end-expiratory pressure (PEEP) was 1–1.5 cm H_2_O. Fraction of inspired oxygen (FiO2) was 1 until 30 min after BD induction, after which FiO_2_ was reduced to 0.5. A frontolateral hole was drilled in the skull and a No. 4 balloon Fogarty catheter (Edwards Lifesciences Co, Irvine, CA) was inserted and inflated in the epidural space. Inflation of the balloon catheter induced initially a characteristic hypotensive period. When MAP returned to its basal level, inflation of the balloon catheter was ceased and anesthesia withdrawn. BD was confirmed by the absence of corneal and pupillary reflexes. Body temperature was maintained at 38°C with a heating pad and MAP was stabilized above 80 mmHg during the BD period. In case of blood pressure drops, Hydroxyethyl starch (HAES-steril 100 g/L, Fresenius Kabi AG, Bad Hamburg, Germany) was administered in a bolus of 0.5 ml with a maximum infusion rate of 1 ml/h. In case of unresponsiveness to HAES, norepinephrine (NA, 0.01 mg/ml, Eu Pharma, Almere, the Netherlands) infusions were added. One hour after BD induction, rats were treated with either 0.9% saline or a low, intermediate or high dose methylprednisolone (40 mg/ml, Pfizer, Capelle aan den IJssel, the Netherlands). Rats were stabilized for 4 h, after which a laparo-thoracotomy was performed. All organs were flushed with 60 ml 0.9% cold saline through the abdominal aorta, after the inferior vena cava was incised for drainage. Lungs were procured and partially snap frozen in liquid nitrogen and partially fixed in formalin and embedded in paraffin.

### RT-qPCR

To assess inflammatory status of donor lungs, gene expression levels were detected by RT-qPCR analyses. TRIzol reagent (Invitrogen Life Technologies, Breda, The Netherlands) was used for isolation of RNA from frozen lung sections. Absence of genomic DNA contamination was verified and integrity was analyzed by gel electrophoresis. Thereafter, cDNA was synthesized according to manufacturer’s instructions. Primer sets used for amplifying fragments of several genes are described in Table 1. Amplification and detection of PCR products were performed by the TaqMan Applied Biosystems 7900HT RT-qPCR system (Applied Biosystems, Foster City, United States), measuring SYBR green (Applied Biosystems) emission. Thermal cycling was performed with a hot start for 2 min on 50°C, followed by 10 min on 95°C. After that, the denaturation step was started with 15 s at 95°C followed by the annealing step and DNA synthesis for 60 s at 60°C, which was repeated 40 times. Melt curve analyses were performed to confirm generation of single, specific amplicons. Samples were analyzed in triplicate and house-keeping gene β-actin was used for normalization of gene expression. Gene expressions were calculated according to the −∆∆CT method (Schmittgen and Livak, 2008).

**TABLE 1:** RT-qPCR primers.

Primer	Gene	Forward primer	Reverse primer	Amplicon (bp)
TNF-α	Tumor necrosis factor-α	AGG​CTG​TCG​CTA​CAT​CAC​TGA​A	TGA​CCC​GTA​GGG​CGA​TTA​CA	67
IL-6	Interleukin-6	CCA​ACT​TCC​AAT​GCT​CTC​CTA​ATG	TTC​AAG​TGC​TTT​CAA​GAG​TTG​GAT	89
IL-1β	Interleukin-1β	CAG​CAA​TGG​TCG​GGA​CAT​AGT​T	GCA​TTA​GGA​ATA​GTG​CAG​CCA​TCT	75
C3	Central complement component 3	CAG​CCT​GAA​TGA​ACG​ACT​AGA​CA	TCA​AAA​TCA​TCC​GAC​AGC​TCT​ATC	96
Cinc-1	Chemokine (C-x-C motif) ligand-1	TGG​TTC​AGA​AGA​TTG​TCC​AAA​AGA	ACG​CCA​TCG​GTG​CAA​TCT​A	78
E-sel	E-selectin	GTC​TGC​GAT​GCT​GCC​TAC​TTG	CTG​CCA​CAG​AAA​GTG​CCA​CTA​C	73
Ccl-2 (Mcp-1)	Chemokine (C-C motif) ligand-2	CTT​TGA​ATG​TGA​ACT​TGA​CCC​ATA​A	ACA​GAA​GTG​CTT​GAG​GTG​GTT​GT	78
IL-10	Interleukin-10	GCA​ACA​GCT​CAG​CGC​ATC​T	ACA​AAC​TGG​TCA​CAG​CTT​TCG​A	71
IL-4	Interleukin-4	CCA​GGG​TGC​TTC​GCA​AAT​T	TTC​ACC​GAG​AAC​CCC​AGA​CTT	76

### Immunohistochemistry

Formalin-fixed paraffin embedded lung sections were stained for myeloperoxidase (MPO) to quantify the number of activated neutrophils, and CD68 to investigate macrophage count. After deparaffinization, antigen retrieval was performed with Tris/HCl 0.1 M and sections were blocked with endogenous peroxidase for 30 min. Thereafter, primary antibody MPO (100 µg/ml Hycult, Uden, The Netherlands) or CD68 antibody (ED-1, 2 µg/ml, Bio-Rad, Lunteren, The Netherlands) was incubated for 1 h. Secondary Goat anti-rabbit (Dako Carpenteria, CA, United States) and rabbit anti-mouse (Dako) horseradish peroxidase (HRP)-conjugated antibodies were incubated for 30 min. Next, tertiary rabbit anti-mouse and goat anti-rabbit HRP-antibodies were incubated for 30 min. All incubations were performed at room temperature and phosphate-buffered saline was used for washing steps. Reaction was developed by 3,3′-diaminobenzidine-peroxidase substrate solution and sections were counterstained with Haematoxylin. ImageJ Software (National Institutes of Health, Bethesda, United States) was used for quantification of cells. Per lung, 10 random snapshots were analyzed on a 400x magnification. Empty or marginal snapshots and snapshots with collapsed or overextended lung tissue were excluded from analyses. Data are presented as mean number of cells/10 snapshots.

### Statistics

Statistical analyses were performed with IBM SPSS Statistics 23 (IBM Corp., Armonk, United States). One-way Analysis of Variance tests were used for multiple comparisons between groups in normally distributed data, followed by post-hoc Bonferroni tests to compare between two groups. In data with skewed distribution, Kruskall-Wallis tests were performed, followed by Mann-Whitney tests to compare between two groups. *p*-values of <0.05 were considered statistically significant. Results are presented as mean ± standard deviation (SD).

## Results

### Chemotaxis and Infiltration of Leukocytes in Lung Tissue of Brain-Dead Donors

**FIGURE 2:**
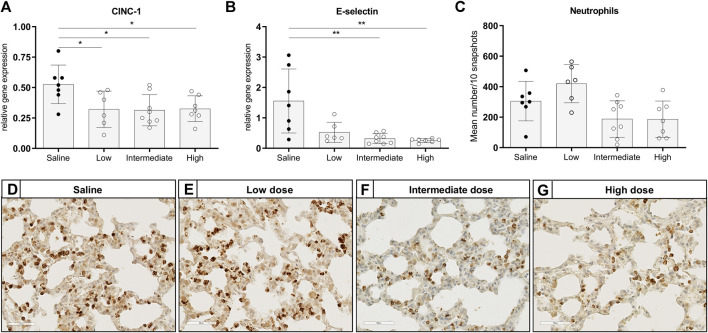
Chemotaxis of neutrophils and infiltration of activated neutrophils in lung tissue. Brain death (BD) was induced in rats assigned to one of four experimental groups, etc. and 1 h after BD induction rats were treated intravenously with 1) 0.9% saline (control) 2) low dose methylprednisolone (5 mg/kg of bodyweight (BW), 3) intermediate dose methylprednisolone (12.5 mg/kg of BW) and 4) high dose methylprednisolone (22.5 mg/kg of BW). mRNA gene expression levels of **(A)** IL8-like CINC-1 and **(B)** E-selectin, involved in chemo-attraction of neutrophils. Data are shown as expressions relative to housekeeping gene β-actin. **(C)** Quantification of activated, MPO-stained neutrophils in lung tissue, presented as mean count per 10 random fields. **(D–G)**: Representative snapshots of lung slides stained for MPO. Values are presented as mean ± SD. *p < 0.05, **p < 0.01.

**FIGURE 3:**
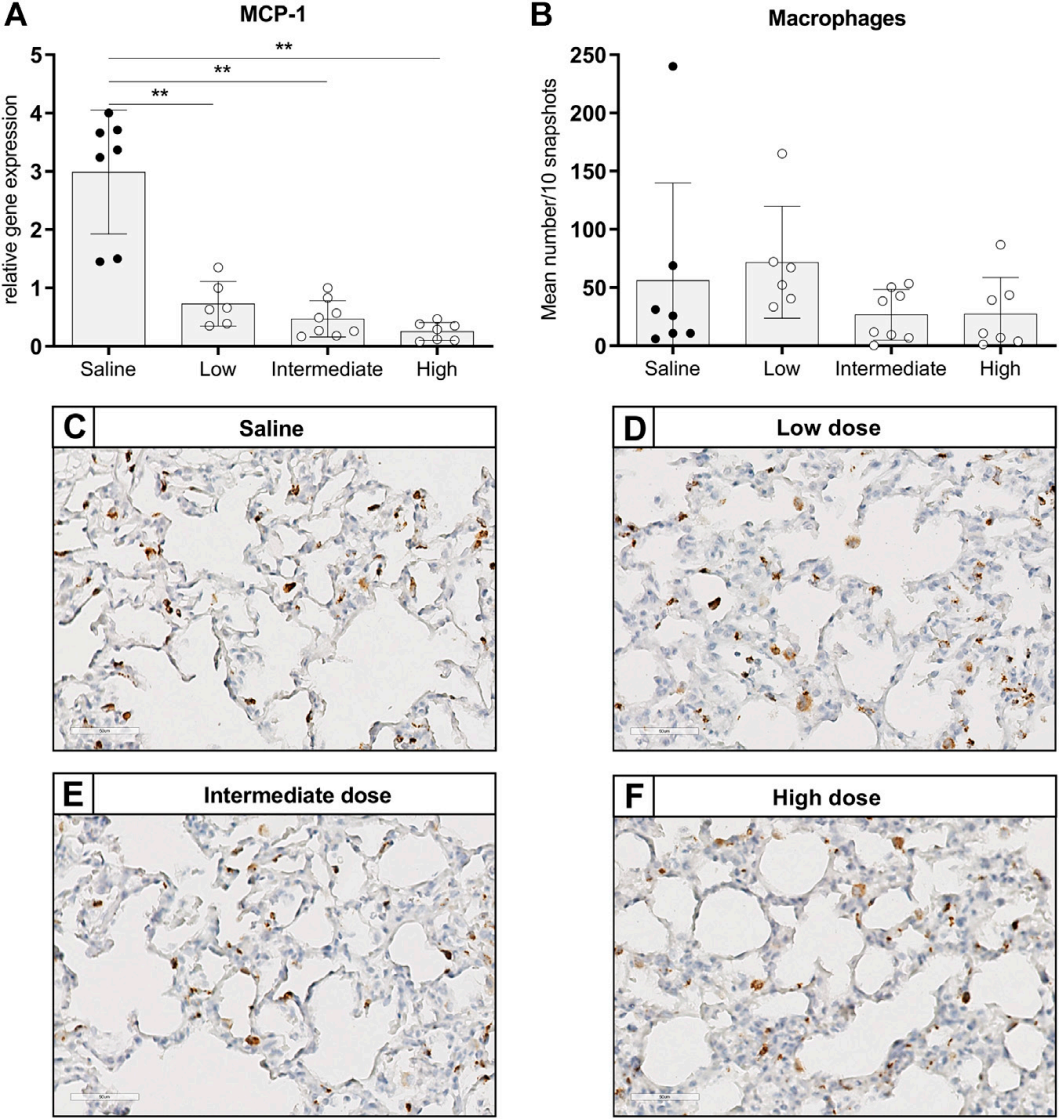
Chemotaxis of macrophages and infiltration of macrophages in lung tissue. Brain death (BD) was induced in rats assigned to one of four experimental groups, etc. and 1 h after BD induction rats were treated intravenously with 1) 0.9% saline (control) 2) low dose methylprednisolone (5 mg/kg of bodyweight (BW), 3) intermediate dose methylprednisolone (12.5 mg/kg of BW) and 4) high dose methylprednisolone (22.5 mg/kg of BW). **(A)** mRNA gene expression levels of MCP-1, involved in chemo-attraction of macrophages. Data are shown as expressions relative to housekeeping gene β-actin. **(B)** Quantification of CD68-stained macrophages in lung tissue, presented as mean count per 10 random fields. **(C–F)**: Representative snapshots of lung slides stained for CD68. Values are presented as mean ± SD. **p < 0.01.

To investigate the effect of different doses methylprednisolone on chemotaxis of leukocytes, we measured gene expression levels of IL-8-like Chemokine (C-x-C motif) ligand 1 (CINC-1), E-selectin and MCP-1. Additionally, we determined the number of activated neutrophils and macrophages in lung tissue. Expression of CINC-1 and E-selectin, involved in chemo-attraction of neutrophils, were down regulated in methylprednisolone treated brain-dead rats (Figures 2A,B). The effect was significant in all groups, except for E-selectin levels in the low dose treatment group. As for influx of activated neutrophils, a decrease was seen in rats treated with an intermediate or high dose methylprednisolone, although significance was not reached (Figures 2C–G). As for macrophages, gene expression levels of MCP-1 showed significant down regulation in all methylprednisolone treated groups (Figure 3A). Nevertheless, the number of infiltrated macrophages was not significantly affected by methylprednisolone treatment on a histological level (Figures 3B–F). Taken together, these results suggest that methylprednisolone treatment reduces gene expressions of chemotactic cytokines for neutrophils and macrophages, primarily in intermediate and high doses.

### Pro-inflammatory Gene Expressions in Lung Tissue of Brain-Dead Donors

**FIGURE 4:**
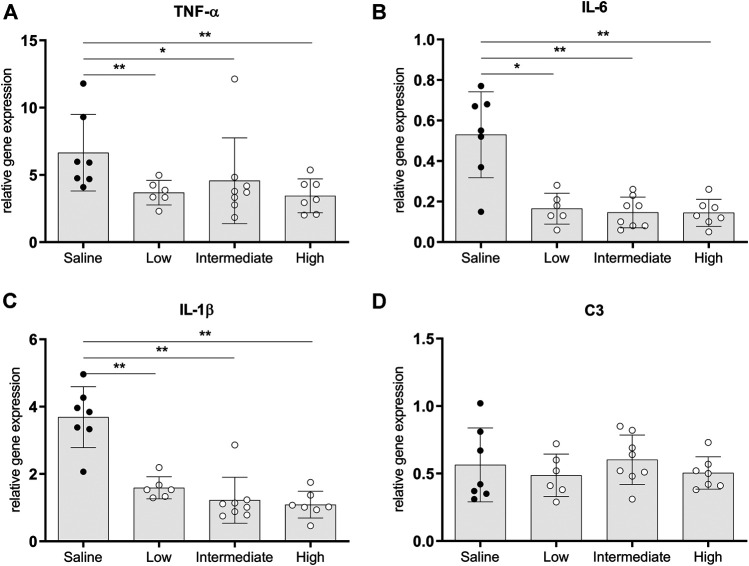
Pro-inflammatory gene expressions in lung tissue. Brain death (BD) was induced in rats assigned to one of four experimental groups, etc. and 1 h after BD induction rats were treated intravenously with 1) 0.9% saline (control) 2) low dose methylprednisolone (5 mg/kg of bodyweight (BW), 3) intermediate dose methylprednisolone (12.5 mg/kg of BW) and 4) high dose methylprednisolone (22.5 mg/kg of BW). mRNA gene expression levels of **(A)** TNF-α, **(B)** IL-6, **(C)** IL-1β and **(D)** C3. Data are shown as expressions relative to housekeeping gene β-actin. Values are presented as mean ± SD. *p < 0.05, **p < 0.01.

The pro-inflammatory state of donor lungs upon treatment with different doses methylprednisolone, was investigated by RT-qPCR analyses in lung tissue. In all methylprednisolone treated groups, gene expressions of TNF-α, IL-6 and IL-1β were attenuated compared to the saline treated control group (Figures 4A–C). Activation of the complement system has previously been described in BD-induced organ damage (Damman et al., 2011). To investigate whether methylprednisolone affected the complement system, we assessed central complement component C3 gene expression levels. No significant differences were seen in C3 gene expression levels between the saline treated control group and the methylprednisolone treated groups (Figure 4D). Collectively, these results indicate that methylprednisolone treatment downregulates pro-inflammatory cytokine expression, but does not involve the complement system.

### Anti-inflammatory Gene Expressions in Lung Tissue of Brain-Dead Donors

**FIGURE 5:**
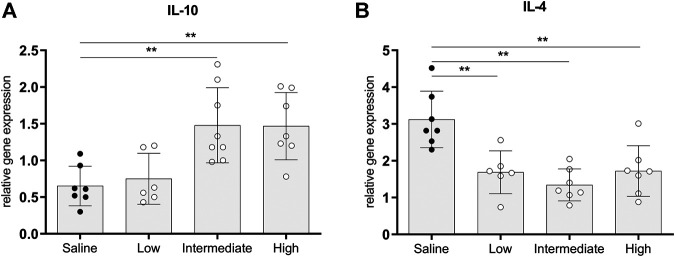
Anti-inflammatory gene expressions in lung tissue. Brain death (BD) was induced in rats assigned to one of four experimental groups, etc. and 1 h after BD induction rats were treated intravenously with 1) 0.9% saline (control) 2) low dose methylprednisolone (5 mg/kg of bodyweight (BW), 3) intermediate dose methylprednisolone (12.5 mg/kg of BW) and 4) high dose methylprednisolone (22.5 mg/kg of BW). mRNA gene expression levels of **(A)** IL-10 and **(B)** IL-4. Data are shown as expressions relative to housekeeping gene β-actin. Values are presented as mean ± SD. **p < 0.01.

Gene expressions of IL-10 and IL-4, traditionally known to be involved in immunoregulating functions, were measured to investigate whether different doses methylprednisolone affects gene expression levels of anti-inflammatory cytokines (Hart et al., 1989; Moore et al., 1993). Gene expression levels of IL-10 in lung tissue were significantly higher in rats treated with intermediate and high doses methylprednisolone, than IL-10 gene expression levels in the saline control group (Figure 5A). In contrast, a decrease in IL-4 expression was seen in all methylprednisolone treated groups (Figure 5B). These results suggest that intermediate and high doses methylprednisolone induce an IL-10 mediated, anti-inflammatory shift in lungs from brain-dead donors.

## Discussion

Methylprednisolone treatment of the potential lung donor is recommended in most clinical protocols, in an attempt to improve lung function and increase organ procurement rates. However, the applied doses vary between centers, which suggests that methylprednisolone treatment guidelines can be optimized in terms of dosing (Dupuis et al., 2014). Dhar et al. suggested that the methylprednisolone dose regimen should be further studied, and investigated the effect of methylprednisolone dose on lung function and organ procurement rate. In the mentioned study, a fixed, low-dose regimen of 300 mg hydrocortisone was compared to a higher dose of 15 mg/kg methylprednisolone in human donors. They showed that the lower dose of 300 mg hydrocortisone led to similar lung function and lung procurement rates, when compared to the higher dose regimen (Dhar et al., 2013). The immunoregulatory effect of methylprednisolone doses on pulmonary immune status was not studied. In this study, we aimed to investigate the effect of three different doses methylprednisolone on the BD-induced inflammatory response, in a rat model for BD. We showed that an intermediate dose of 12.5 mg/kg methylprednisolone reduces BD-induced lung inflammation and additionally promotes a protective, anti-inflammatory response.

We first studied the effect of different doses methylprednisolone on chemotaxis of leukocytes on a gene expression level. All three doses methylprednisolone reduced gene expression levels of potent chemo-attractants for neutrophils and macrophages, reflected by lower IL-8-like CINC-1, E-selectin and MCP-1 gene expression levels. Nevertheless, no significance was reached in reduced neutrophil count, although a trend was observed upon treatment with intermediate and high doses methylprednisolone. Earlier studies investigated an even higher treatment dose of 30 mg/kg methylprednisolone in brain-dead rats. Also in these studies, the amount of neutrophils was not affected by methylprednisolone treatment, even when methylprednisolone was administered shortly after BD induction (Pilla et al., 2013; Felipe Lopes Araujo et al., 2014). Based on these observations, we speculate that methylprednisolone treatment minimally affects donor-derived neutrophils, which are immediately attracted to the site of damage upon BD. However in contrast, reduced neutrophil chemo-attractants may have a forthcoming beneficial effect on recipient-derived neutrophil infiltration, as described by Paulus *et al.* In the mentioned study, lungs were flushed with 12 mg/kg methylprednisolone before preservation, which reduced neutrophil infiltration after lung transplantation (Paulus et al., 2013).

Along with the amount of neutrophils in lung tissue, macrophage count was not affected upon treatment with different doses methylprednisolone. However, the present tissue macrophages may shift from a cytotoxic (M1) to a more anti-inflammatory (M2) phenotype, which contributes to a downregulating effect on pro-inflammatory response. We first studied the effect of methylprednisolone on pro-inflammatory mediators, which may be downregulated through inhibition of NF-κB (Rhen and Cidlowski, 2005). Indeed, in line with previous studies in which 30 mg/kg methylprednisolone was administered, we show a strong, downregulating effect of all doses methylprednisolone on TNF-α, IL-6 and IL-1β gene expressions, cytokines that may be secreted by M1 macrophages (Pilla et al., 2013; Felipe Lopes Araujo et al., 2014). In addition, glucocorticoids such as methylprednisolone are described to stimulate polarization from M1 to the M2c subset of M2 macrophages, which are described to secrete the anti-inflammatory cytokine IL-10 (John et al., 1998; Roszer, 2015). Our results are in line with previously described immunoregulatory effects of methylprednisolone through IL-10, although an upregulation of IL-10 was only observed in rats treated with intermediate and high doses methylprednisolone (Park-Min et al., 2005; Paulus et al., 2013). Hence, intermediate and high doses methylprednisolone may stimulate M2 polarization through the M2c subset of M2 macrophages. In addition, Paulus *et al.* described an upregulation of IL-4 expression upon methylprednisolone treatment. In the mentioned study, the effect of methylprednisolone pretreatment on the immune response was investigated, measured at 48 h after lung transplantation (Paulus et al., 2013). The cytokine IL-4 is described to stimulate polarization to the M2a subset of M2 macrophages, which may subsequently lead to secretion of the anti-inflammatory cytokine IL-10 (Roszer, 2015). However in our study, IL-4 expression was decreased in all methylprednisolone treated groups, when compared to the saline treated control group. The controversial effects of corticosteroids on IL-4 expression have previously been illustrated, and are suggested to be the result of direct *vs.* indirect effects of corticosteroids on target cells (Daynes and Araneo, 1989; Wu et al., 1991; Masuyama et al., 1994; Snijdewint et al., 1995; Ramírez et al., 1996; Gratchev et al., 2008). On a short term, corticosteroids might directly inhibit cytokine synthesis in both Th1 and Th2-cells, such as IL-4. However on a longer term, corticosteroids downregulate IL-12 production by macrophages, which might indirectly stimulate IL-4 production (Blotta et al., 1997). Our study represents a snapshot at only 4 h after induction of BD. Therefore, we speculate that the results on IL-4 expression in our study reflect a short-term, inhibiting effect of methylprednisolone, and M2 polarization through the M2a subset is thereby not evident in our model.

The BD-induced inflammatory response in donor lungs is an important target, since the postoperative inflammatory status in transplanted lungs is described to be associated with long-term outcome (Hall et al., 2012). Nevertheless, despite that methylprednisolone treatment is recommended in various donor management protocols, the benefit of methylprednisolone has not yet been irrefutably proven. Duipuis et al. concluded in their review that the current clinical evidence is conflicting and of poor quality, which is partially due to the lack of homogeneous data. In addition, interacting factors such as simultaneous administration of other hormonal therapies, complicate research on the clinical effect of corticosteroids in donor management. For that reason, we emphasize the importance of fundamental research in the search for the significance of methylprednisolone treatment on BD-induced injury. We consider our rat model for BD as the strong point of our study, which allows us to investigate the effect of different doses methylprednisolone on BD-induced lung damage alone. Based on our results, we consider the intermediate dose of 12.5 mg/kg methylprednisolone as most suitable to target BD-induced lung inflammation, since both the pro-inflammatory response was reduced and an additional anti-inflammatory response was promoted. The high dose of 22.5 mg/kg showed no additional beneficial effect, which suggests that the maximum effect has been achieved with an intermediate dose methylprednisolone. Besides that, possible negative effects of high doses methlyprednisolone should be carefully considered. High doses methylprednisolone might dysregulate blood glucose levels in organ donors, which negatively effects organ retrieval rate and graft function (Blasi-Ibanez et al., 2009; Sally et al., 2014). In addition, the effect of methylprednisolone on other potential donor organs should be taken into account. Rebolledo et al. studied the effect of different doses methylprednisolone on the quality of donor livers and kidneys, and showed that a high dose of 22.5 mg/kg showed no additional beneficial effect compared to an intermediate dose of 12.5 mg/kg methylprednisolone. Thereby, 22.5 mg/kg methylprednisolone even correlated with higher liver tissue injury markers, which possibly reflects reduced quality of the donor liver (Rebolledo et al., 2015).

The recommended methylprednisolone dose of 12.5 mg/kg for rats in our study, might be close to doses previously investigated in human studies. However, it should be noted that extrapolation of the optimal treatment dose from rats to humans should be carefully performed, given the biological differences between species. Scaling of dose based on body weight might be insufficient, due to differences in body surface area and pharmacokinetics (Reigner and Blesch, 2002). In a retrospective study by Folette et al., human donors subjected to 14.5 mg/kg methylprednisolone were compared to untreated controls. The authors concluded that methylprednisolone treatment of the organ donor led to better oxygenation ratios and higher procurement rates (Follette et al., 1998). In addition, Nath et al. showed increased lung procurement rates when donors were treated with 15 mg/kg methylprednisolone (Nath et al., 2010). In a randomized-controlled study, Venkateswaran et al. compared donor treatment with 15 mg/kg methylprednisolone to untreated controls, etc. and found less pumonary edema and higher suitability of donor lungs, despite no differences in oxygenation ratio (Venkateswaran et al., 2008). More recently, Dhar et al. compared donor treatment of 15 mg/kg methlyprednisolone to a fixed, low dose of 300 mg hydrocortisone, which corresponds to a dose of <1 mg/kg methylprednisolone for a 70-kg donor. In the mentioned study, they did not find differences between lung procurement rates or donor lung function, suggesting that lower corticosteroid doses might be sufficient in donor management (Dhar et al., 2013). Yet, the beforementioned studies did not specifically focus on the effect of methylprednisolone dose on pulmonary inflammation elicited by the pathophysiology of BD. Nevertheless, speculations may be drawn from other disease models, such as acute respiratory stress syndrome (ARDS). Early ARDS is characterized by diffuse alveolar injury, lung edema and neutrophil-mediated inflammation, which resembles the pathophysiology of BD-induced lung damage. An early study by Bernard et al. in 1987 showed no improvement in mortality or ventilatory characteristics when patients were treated with high doses methylprednisolone of 30 mg/kg every 6 h, possibly due to suppression of HPA axis and higher risk of infection (Bernard et al., 1987). A recent retrospective and observational study confirmed that high dose methylprednisolone may not be suitable for patients with ARDS, by showing increased mortality rates in ARDS patients treated with high dose methylprednisolone (defined as >500 mg methylprednisolone/day) compared to the non-high-dose group (Kido et al., 2018). In contrast, studies investigating low-dose methylprednisolone (1 mg/kg/day) showed reduced mortality and duration of mechanical ventilation in patients with early ARDS, after treatment for several weeks (Meduri et al., 2007). In addition, patients with unresolving ARDS treated with 2 mg/kg/day methylprednisolone showed obvious reductions in TNF-a, IL-1β and IL-6 serum levels, after 4 weeks of treatment (Meduri et al., 1998). However, the effect of low-dose methlyprednisolone treatment on anti-inflammatory cytokines was not addressed. Whether low doses of 1–2 mg/kg methylprednisolone are sufficiently adequate to both downregulate the pro-inflammatory response and stimulate an anti-inflammatory response in human brain-dead donors as observed in our rat model, requires further clinical investigation. In addition, it should be noted that the beforementioned doses in the treatment of ARDS are administered over several weeks, while the time frame of donor mangement is closer to several hours or days.

Data from laboratory animal models can be of exceptional value, since pathophysiological mechanisms may be difficult to test in humans, while animal models provide more experimental possibilities. Nevertheless, even though BD-induced lung damage and inflammation were reliably reprodcued in our rat model, it is impossible to reproduce all the characteristics of the human lung transplantation process in an animal model. The whole chain of events in human lung donation- and transplantation is highly complex due to many confounding factors. In our study we did not investigate the effect of different doses methylprednisolone on ischemia-reperfusion injury, since transplantation of donor lungs was not included in our model. Hence, the possible longer-term immunomodulatory effects of different doses methylprednisolone, in presence of circulating recipient-derived leukocytes, requires attention in future studies.

This study provides a foundation for further research on methylprednisolone regimens and BD-induced lung inflammation. We conclude from this study that an intermediate dose of 12.5 mg/kg methylprednisolone is the optimal treatment dose for BD-induced lung inflammation in rats, which reduces the pro-inflammatory state and additionally promotes a protective, anti-inflammatory response.

## Data Availability

The raw data supporting the conclusions of this article will be made available by the authors, without undue reservation.
